# Involvement of Histidine Residue His382 in pH Regulation of MCT4 Activity

**DOI:** 10.1371/journal.pone.0122738

**Published:** 2015-04-28

**Authors:** Shotaro Sasaki, Masaki Kobayashi, Yuya Futagi, Jiro Ogura, Hiroaki Yamaguchi, Ken Iseki

**Affiliations:** 1 Laboratory of Clinical Pharmaceutics & Therapeutics, Division of Pharmasciences, Faculty of Pharmaceutical Sciences, Hokkaido University, Kita-12-jo, Nishi-6-chome, Kita-ku, Sapporo 060–0812, Japan; 2 Department of Pharmacy, Hokkaido University Hospital, Sapporo 060–8648, Japan; University of Minho, PORTUGAL

## Abstract

Monocarboxylate transporter 4 (MCT4) is a pH-dependent bi-directional lactate transporter. Transport of lactate *via* MCT4 is increased by extracellular acidification. We investigated the critical histidine residue involved in pH regulation of MCT4 function. Transport of lactate *via* MCT4 was measured by using a *Xenopus laevis* oocyte expression system. MCT4-mediated lactate transport was inhibited by Zn^2+^ in a pH physiological condition but not in an acidic condition. The histidine modifier DEPC (diethyl pyrocarbonate) reduced MCT4 activity but did not completely inactivate MCT4. After treatment with DEPC, pH regulation of MCT4 function was completely knocked out. Inhibitory effects of DEPC were reversed by hydroxylamine and suppressed in the presence of excess lactate and Zn^2+^. Therefore, we performed an experiment in which the extracellular histidine residue was replaced with alanine. Consequently, the pH regulation of MCT4-H382A function was also knocked out. Our findings demonstrate that the histidine residue His382 in the extracellular loop of the transporter is essential for pH regulation of MCT4-mediated substrate transport activity.

## Introduction

Monocarboxylate transporter 4 (MCT4) is an H^+^ gradient-dependent lactate transporter [1]. The transporter is identical to solute carrier family 16, member 3 (SLC16A3), the function of which has been determined and which has been shown to have a low affinity for lactate compared to other lactate carriers [1–4].

It has been shown that critical functional positions within MCT4 include the amino acid residue involved in lactate recognition, but the detailed pH regulation mechanism has not yet been determined [1]. Previous reports for other H^+^/substrate co-transporters suggest important roles for histidine residues [5]. Natural resistance-associated macrophage protein 2 (Nramp2/SLC11A2) is a pH-dependent divalent metal transporter with broad substrate specificity including Fe^2+^, Mn^2+^, Co^2+^, Cd^2+^, Cu^2+^, Ni^2+^, Pb^2+^, and possibly Zn^2+^ and may function by an H^+^ cotransport mechanism [6]. His267/His272 are not directly involved in metal binding but, rather, play an important role in pH regulation of metal transport by Nramp proteins [7]. The human H^+^-coupled folate transporter PCFT (SLC46A1) is involved in the transport of folates across the apical membranes of enterocytes within the acidic microclimate at the absorptive surface of the proximal jejunum [8]. His281 is not essential for proton coupling to the carrier [9]. H^+^-coupled oligopeptide transporter PEPT1 was assigned to the solute carrier family 15 as SLC15A1 [10]. Transport activity of PEPT1 is inhibited by diethyl pyrocarbonate (DEPC), and His57 of PEPT1, located at or near the substrate binding site, is involved in the pH regulation of transport activity [11]. DEPC is known to suppress the H^+^ acceptor or donor function of histidine residues of proteins. The transport activity of human H^+^-coupled amino acid transporter PAT1 (SLC36A1) is also inhibited by DEPC, and it was shown that a histidine residue is important for the transport activity of PAT1 [12, 13].

MCT4 is composed of 465 amino acid residues and is predicted to contain 12 transmembrane domains. This transporter has five histidine residues, one in the extracellular loop, another in the intracellular loop and the other three in the carboxy terminal facing the cytosol [1]. The present study, using chemical modification by DEPC and site-directed mutagenesis, was carried out to confirm that histidine residues are involved in the pH regulation of MCT4 activity. There has been no mutational study of histidine residues in MCT4. Our investigation showed that His382 is essential for the regulation of pH-sensitive transport activity of the transporter.

## Methods

### Synthesis of cRNA

Molecular cloning of MCT4 and MCT1 was performed as previously described [1, 14]. Plasmids were linearized with *Not*I (MCT1) or *Xho*I (MCT4), and MCT1 and MCT4 cRNAs were synthesized *in vitro* using T7 RNA polymerase (Epicentre) from these cDNAs.

### Site-directed mutagenesis

An MCT4 mutant was obtained by using the Quickchange protocol (Agilent). The mutagenic oligonucleotides used were 5’-GATGCGACCGCCGTCTACATGTACGTGTTC-3’ for the forward primer and 5’-CATGTAGACGGCGGTCGCATCCAGGAGTTTG-3’ for the reverse primer, which replace a histidine residue at position 382 with alanine (MCT4-H382A). A High Pure Plasmid Isolation Kit (Roche) was used to isolate the plasmid DNA. Mutation was confirmed by DNA sequencing using a BigDye Terminator v3.1 Cycle Sequencing Kit from Applied Biosystems.

### Uptake measurements in MCT1 cRNA or MCT4 cRNA-injected oocytes

*Xenopus laevis* oocytes were collected under anesthesia (immersion in a solution of 1 g/l MS-222) from toads. Stage IV or V oocytes from *Xenopus laevis* were isolated and maintained at 17°C in Barth’s solution as previously described [1]. Oocytes were injected with 50 ng cRNA in a 50-nl volume and incubated for 3–6 days. The transport functions of heterologously expressed MCT1 and MCT4 were monitored. The transport buffer used in this study was a standard buffer (100 mM NaCl, 2 mM KCl, 1 mM MgCl_2_, 1 mM CaCl_2_, and 10 mM Good’s buffer). HEPES was used for pH 8.0–7.0 buffer, and MES was used for pH 7.0–5.5 buffer. We measured the uptake of radiolabeled compounds (L-lactate [^14^C: 0.1 μCi/ml], nicotinic acid [^3^H: 0.5 μCi/ml] and salicylic acid [^14^C: 0.1 μCi/ml]) by using a liquid scintillation counter. Uptake of a radiolabeled compound in water-injected and cRNA-injected oocytes was performed, and oocytes were washed with ice-cold transport buffer as previously described [1]. Metal inhibition of 0.1 mM lactate uptake by oocytes expressing MCT4-WT, MCT4-H382A and MCT1 and water-injected oocytes was measured in the presence or absence of 5 mM of each metal. The oocytes were incubated with metals and [^14^C] lactate for 10 min. We performed the experiments using DEPC and hydroxylamine in conformity to the method of Baird *et al*. [15]. All data are given as means±S.E. of three independent experiments, and 12–15 oocytes were used in each of the experiments on uptake of radiolabeled compounds. Student’s *t* test was used to determine the significance of differences between the means of two groups. Statistical significance among means of more than two groups was analyzed by one-way analysis of variance (ANOVA). *p*<0.05 was considered statistically significant.

### Materials

[^14^C] L-lactate, [^3^H] nicotinic acid and [^14^C] salicylic acid were obtained from American Radiolabeled Chemicals. *Xenopus laevis* toads were obtained from Hokudo. Permission for this study was obtained from the Committee on Animal Experimentation, Hokkaido University. All other chemicals used were of the highest purity available.

## Results

### Effects of metals on transport activity of MCT4

**Fig 1:**
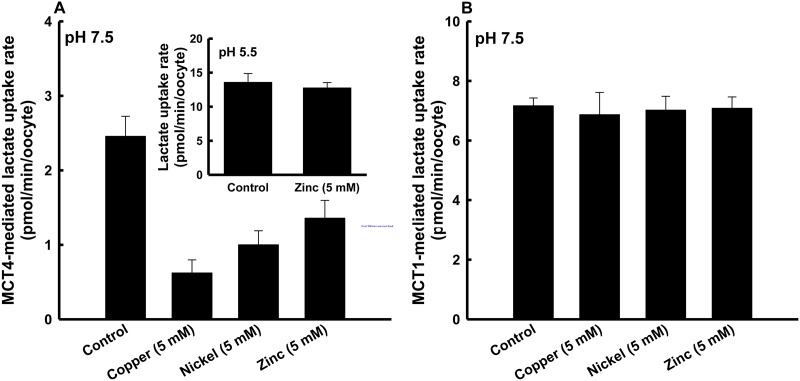
Inhibitory effects of metals on MCT4-mediated lactate uptake. (A) MCT4-mediated uptake of 0.1 mM lactate (0.1 μCi/ml) was measured in a transport buffer of pH 7.5 containing 5 mM of each metal for 10 min. The inset shows the effect of 5 mM zinc under an acidic condition (pH 5.5). (B) MCT1-mediated uptake of 0.1 mM lactate (0.1 μCi/ml) was measured in a transport buffer of pH 7.5 containing 5 mM of each metal for 10 min. The background uptake values of water-injected oocytes were subtracted. Data are presented as means±S.E. of three experiments.

We investigated the transport of lactate *via* MCT4 using a heterologous functional expression system. We expressed cloned MCT4 in *Xenopus laevis* oocytes by injection of cRNA. First, we examined that effect of 5 mM of each metal on MCT4-mediated transport of lactate at pH 7.5. The transport of lactate (0.1 mM) *via* MCT4 was markedly inhibited by the metals with inhibitory effects in the order of copper > nickel > zinc (Fig 1A). In contrast, MCT1-mediated lactate transport was not inhibited by the metals (*p*>0.05) (Fig 1B). We also investigated the inhibitory effect of zinc at pH 5.5 on the transport of lactate by MCT4. Under this condition, we could not detect interaction of zinc with MCT4 (*p*>0.05) (Fig 1A: inset). The metals combined with an amino acid residue(s), and the p*K*_a_ of this residue is between 5.5 and 7.5. This value accords with p*K*_a_ of the histidine residue having an imidazole group of its side chain. These results imply that the metals combined with a histidine residue of MCT4.

### Effect of a histidine residue modifier, DEPC, on lactate transport through MCT4-expressing oocytes

**Fig 2:**
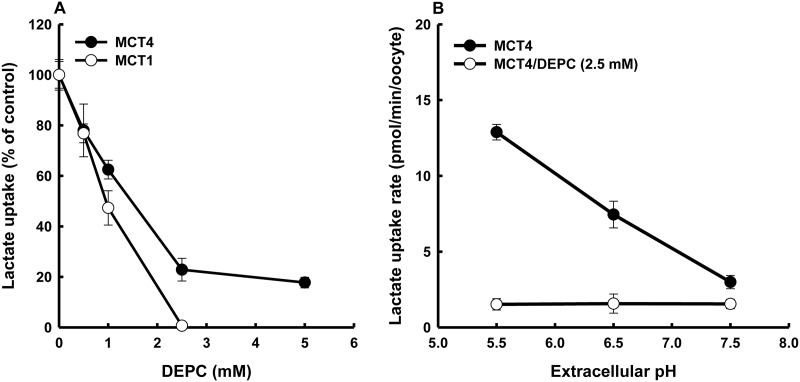
Inhibitory effects of DEPC on MCT4-mediated uptake of lactate. (A) Oocytes expressing MCT4 and MCT1 were washed and incubated at 25°C with DEPC at different doses. After 10 min, the oocytes were washed twice and incubated with a transport buffer (pH 5.5) containing 0.1 mM lactate (0.1 μCi/ml) for 10 min. (B) Oocytes were subjected to a 10-min pre-incubation in a transport buffer (pH 7.5) containing 2.5 mM DEPC at 25°C. The oocytes were washed and uptake of 0.1 mM lactate (0.1 μCi/ml) was measured for 10 min at the pH values indicated. Values for water-injected oocytes were subtracted from the values for MCT4-expressing oocytes. Each value is the mean±S.E. of three experiments.

**Fig 3:**
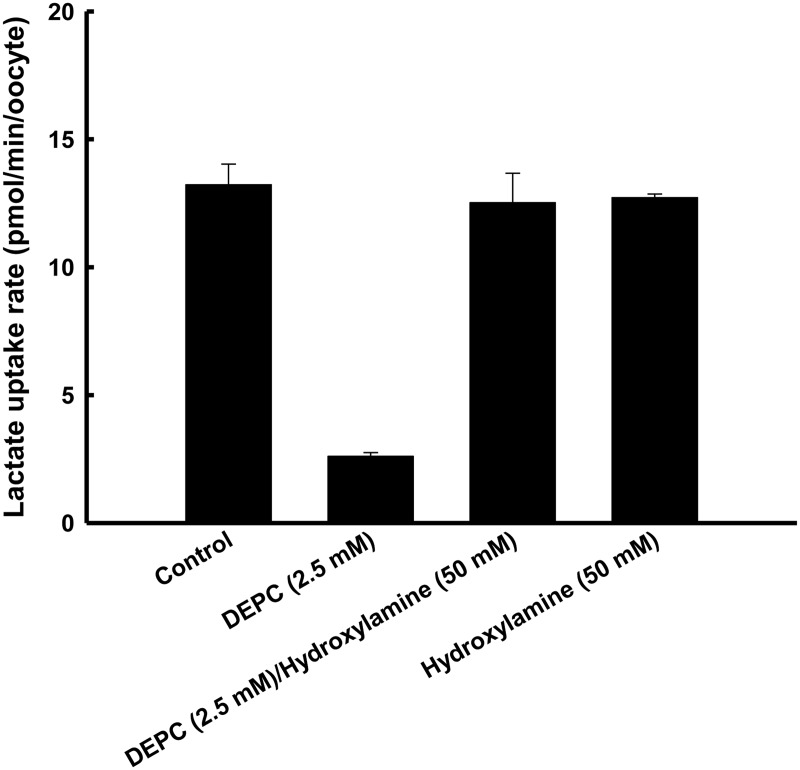
Determination of the kind of DEPC-sensitive residue. Oocytes were pre-incubated for 10 min in a transport buffer in the presence or absence of DEPC (2.5 mM). After DEPC treatment, the oocytes were additionally incubated for 1 h in a transport buffer in the presence or absence of hydroxylamine (50 mM). The oocytes were incubated for 10 min in a transport buffer of pH 5.5 containing 0.1 mM lactate (0.1 μCi/ml). Data are presented as means±S.E. of three experiments. MCT4-specific uptake was calculated by subtracting the uptake in water-injected oocytes from the uptake in MCT4 cRNA-injected oocytes.

DEPC is considered to be specific for histidine within the pH range of 5.5–7.5 [16] as was used in this study. Lactate transport *via* MCT4 under the condition of DEPC treatment was decreased substantially but did not completely disappear (Fig 2A). The *K*_0.5_ for inactivation of DEPC was approximately 1.5 mM for MCT4 with DEPC treatment time of 10 min. On the other hand, DEPC treatment completely abolished the transport activity of MCT1. As shown in Fig 2B, MCT4 clearly showed pH-sensitive transport activity that increased with increase in extracellular proton concentration, whereas the pH sensitivity of MCT4 was voided by exposure to DEPC. Additionally, we tested the effect of hydroxylamine to determine whether the effects of DEPC would be reversed. Treatment with 50 mM hydroxylamine for 1 h resulted in recovery from the inactivation of DEPC (Fig 3). DEPC and zinc also react with cysteine residues in a protein under certain conditions, though we previously reported that the cysteine-modifying agent 5,5’-dithio-bis(2-nitrobenzoic acid) (DTNB) has no effect on MCT4-mediated lactate transport [1]. These results indicated that the DEPC-modified histidine residue is an H^+^ binding site and extracellular pH sensor.

### Effects of monocarboxylates on the DEPC modification of MCT4

**Fig 4:**
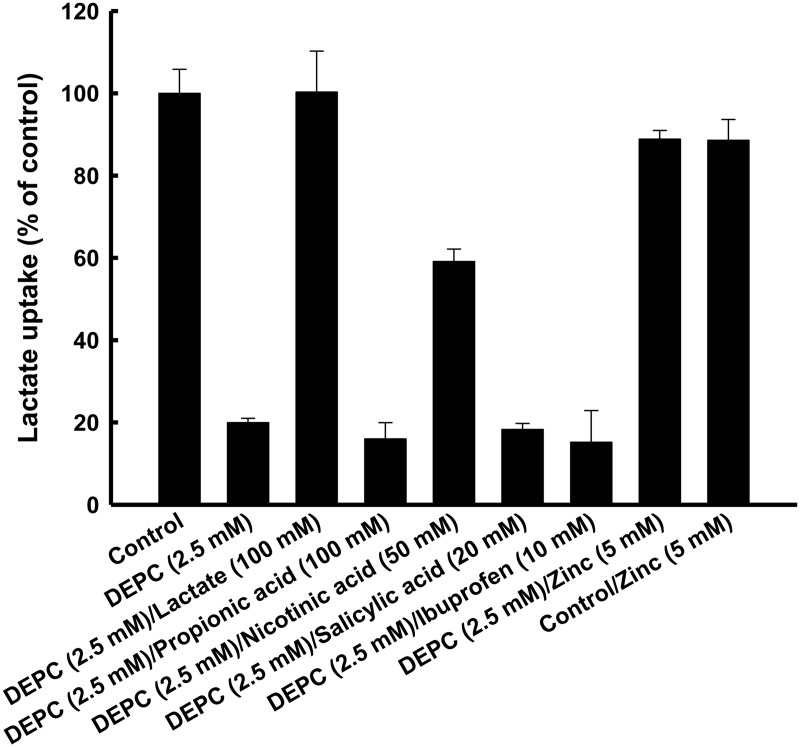
Effects of various compounds on DEPC modification. Oocytes were pre-incubated for 10 min in a transport buffer (pH 7.5) containing 2.5 mM DEPC at 25°C in the absence or presence of 100 mM lactate, 100 mM propionic acid, 50 mM nicotinic acid, 20 mM salicylic acid, 10 mM ibuprofen and 5 mM zinc. After treatment with DEPC, the oocytes were rinsed twice with a transport buffer not including a radiolabeled compound. Oocytes were additionally incubated twice for 5 min each time with the transport buffer. The oocytes were incubated for 10 min in a transport buffer of pH 5.5 containing 0.1 mM lactate (0.1 μCi/ml). Data are presented as means±S.E. of three experiments. MCT4-specific uptake was calculated by subtracting the uptake in water-injected oocytes from the uptake in MCT4 cRNA-injected oocytes.

**Fig 5:**
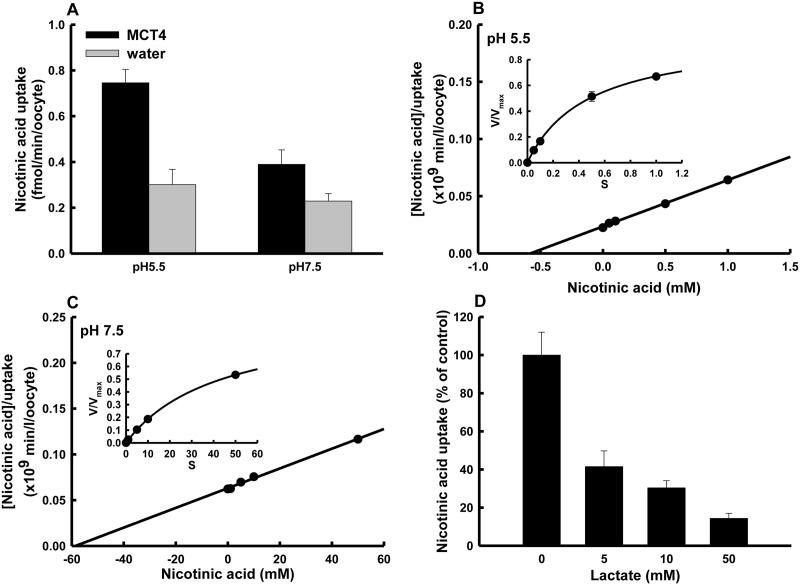
Nicotinic acid transport *via* MCT4. (A) MCT4 cRNA-injected or water-injected oocytes were incubated for 5 min at 25°C with 10 nM nicotinic acid (0.5 μCi/ml) at pH 5.5 or 7.5. Data are presented as means±S.E. of three experiments. Uptake of nicotinic acid was measured with 5-min incubation at 25°C in a transport buffer of pH 5.5 (B) or pH 7.5 (C) in the presence of an increasing dose of nicotinic acid. The background uptake values of water-injected oocytes were subtracted. Only the MCT4-specific uptake was used for kinetic analysis. Results are Hanes plots for which each value is the mean value for three experiments, and lines were fitted using the least-squares method: from each line, *K*_m_ was estimated as the *x*-axis intercept. (D) Dose dependency of MCT4-mediated lactate inhibition of nicotinic acid uptake. The nicotinic acid uptake was measured at pH 5.5 for 5 min. Data are presented as means±S.E. of three experiments.

Since the histidine residue might have been located in the proton binding site, we investigated the effects of monocarboxylates on the DEPC modification of MCT4. Fig 4 shows the effects of lactate, propionic acid, nicotinic acid, salicylic acid and ibuprofen on DEPC inactivation of lactate uptake *via* MCT4. It is known that lactate and nicotinic acid are taken up by MCT4. Additionally, propionic acid and ibuprofen possess the propionic acid structure as well as lactate. The structures of salicylic acid and nicotinic acid are also very similar. The inhibitory effect of DEPC on MCT4-mediated transport activity was prevented by lactate and nicotinic acid but not by propionic acid, salicylic acid or ibuprofen. These results indicated that nicotinic acid, but not salicylic acid, is a potential substrate of MCT4. To elucidate the binding site of zinc, we also investigated the effect of zinc on DEPC treatment of MCT4. The DEPC-induced suppression of lactate uptake *via* MCT4 was also prevented by zinc. This result indicated that lactate, nicotinic acid and zinc bind to the same histidine residue as that involved in regulation of pH-dependent activity by MCT4. We investigated the transport of nicotinic acid and salicylic acid, the structures of which are very similar, *via* MCT4 (Table 1). The uptake of nicotinic acid was greater in cRNA-injected oocytes than in water-injected oocytes. In contrast, cRNA-induced salicylic acid uptake activity was not detected. We next investigated the effect of extracellular pH on nicotinic acid transport *via* MCT4 (Fig 5A). The uptake of nicotinic acid by MCT4-expressing oocytes was reduced by alkalizing the extracellular pH, indicating that nicotinic acid uptake *via* MCT4 involves a proton-linked transport system. The uptake of lactate was decreased by alkalization of the extracellular pH, and the *K*_m_ values showed a pH dependency [1]. Thus, a dose-dependency study was performed to test the pH-dependent affinity of MCT4 for nicotinic acid (Fig 5B and 5C). MCT4 showed a much lower affinity for nicotinic acid in an alkaline condition than in an acidic condition. The affinities were found to be in the following order: pH 5.5 (0.57 mM) > pH 7.5 (57 mM). Fig 5D shows the effect of lactate as a classical substrate on nicotinic acid uptake *via* MCT4. Lactate inhibited nicotinic acid uptake in a concentration-dependent manner. This experiment was performed when radiolabeled nicotinic acid and non-labeled lactate were added at the same time to the reaction.

**Table 1:** Uptake of nicotinic and salicylic acids in MCT4 and MCT1 cRNA-injected oocytes and water-injected oocytes

	Uptake (pmol/min/oocyte)		
Compound	MCT4 cRNA-injected oocytes	MCT1 cRNA-injected oocytes	water-injected oocytes
**Salicylic acid (100 μM)**	**7.7±0.3**	**10.5±0.4**	**10.8±1.0**
**Nicotinic acid (100 μM)**	**7.7±0.2**	**19.3±0.9**	**3.9±1.2**

Uptake of salicylic acid and nicotinic acid (100 μM: labeled + unlabeled) in MCT4 and MCT1 cRNA-injected oocytes and water-injected oocytes was measured with 5-min incubation at 25°C in a transport buffer of pH 5.5. Data are presented as means±S.E. of three experiments.

### The pH-sensitivity of lactate transport via MCT4 mutant

**Fig 6:**
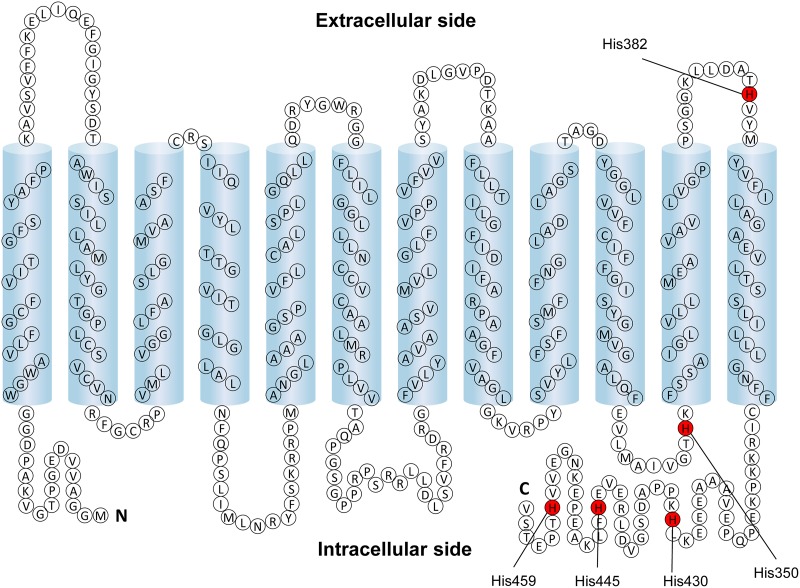
Putative topology of human MCT4. The histidine residues of MCT4 are shown in red.

**Fig 7:**
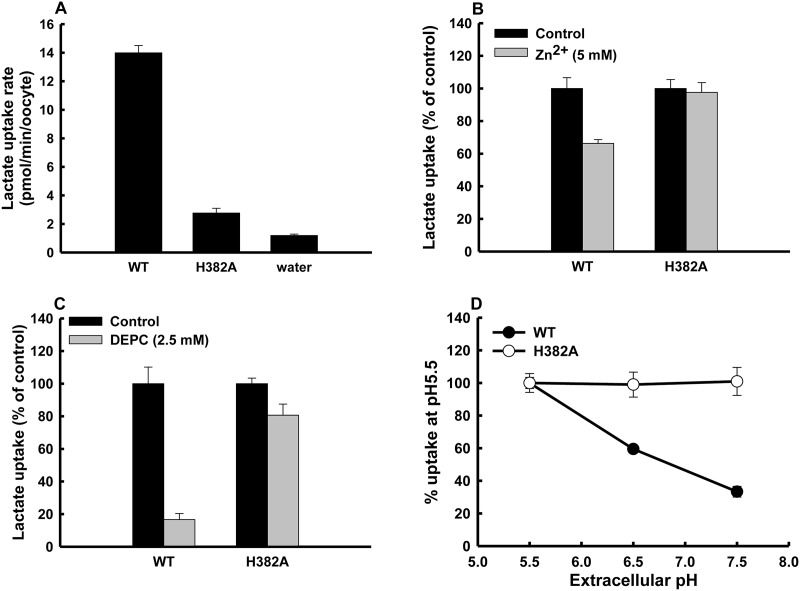
Transport of lactate *via* MCT4-mutant. (A) Oocytes injected with MCT4-WT cRNA, MCT4-H382A cRNA, or water were incubated with 0.1 mM lactate (0.1 μCi/ml) for 10 min. (B) Sensitivities against 5 mM zinc of lactate transport activities *via* MCT4 with a histidine residue mutant. Uptake of 0.1 mM lactate (0.1 μCi/ml) was measured at pH 7.5 for 10 min. (C) Wild-type and histidine mutant MCT4 cRNA-injected oocytes were incubated for 10 min with/without 2.5 mM DEPC in a buffer of pH 7.5 at 25°C. Uptake of 0.1 mM lactate (0.1 μCi/ml) was measured for 10 min at pH 5.5. (D) pH regulation of lactate uptake *via* wild-type and mutant MCT4. Uptake of 0.1 mM lactate was measured for 10 min at the indicated pH by oocytes expressing proteins of interest. Values are presented as percentages of uptake measured at pH 5.5. All data are presented as means±S.E. of three experiments.

The complete abolishment of pH dependency of MCT4 by DEPC treatment indicated that a histidine residue plays an important role in the regulation of activity. However, the position of the histidine residue is not known. To address this issue, based on a putative topology model of MCT4 (Fig 6) [1], we mutated the amino acid residue that might be involved in extracellular pH dependency. This protein has five histidine residues, one in the extracellular loop, another in the intracellular loop and the other three in the carboxy terminal facing the cytosol. DEPC cannot react with histidine residues located in the intracellular side within proteins because it is membrane-impermeable [16]. Hence, we considered that the histidine residue located in the extracellular loop is important for the pH dependency. The activity of MCT4-H382A was weak compared with that of the wild type (Fig 7A). These results imply that the sensitivity of the mutant for lactate and/or proton was decrease compared with that of the wild type. We then investigated the kinetic characteristic of lactate transport *via* MCT4-H382A. The transport of lactate *via* the mutant was saturated at high concentrations of lactate. The kinetic parameters at pH 5.5 were calculated by nonlinear regression of the Michaelis-Menten equation, and *K*_m_ and *V*_max_ values were estimated to be 3.3±0.3 mM and 55±1.6 pmol/min/oocyte, respectively (Table 2). The *K*_m_ value was similar to a previously reported value for MCT4-WT-expressing oocytes [1]. Fig 7B shows the inhibitory effects of zinc on lactate uptake *via* MCT4-WT and MCT4-H382A. Zinc inhibited lactate uptake in WT-expressing oocytes, but we could not detect any effect of zinc on lactate uptake in the mutant-expressing oocytes (*p*>0.05). We then investigated the effects of chemical modification of histidine by DEPC treatment on MCT4-WT and MCT4-H382A-mediated lactate transport (Fig 7C). DEPC treatment greatly suppressed the transport activity of MCT4-WT. However, we could not detect a suppressive effect of DEPC treatment on the transport activity of MCT4-H382A (*p*>0.05). These results indicated that His382 is a zinc and DEPC-binding site and that this residue plays a crucial role in MCT4 activity. The pH dependency of MCT4-H382A was similar to that of MCT4-WT under the DEPC treatment condition (Fig 7D and Table 3). These observations revealed that the extracellular loop histidine residue His382 located at least near the substrate recognition site contributed markedly to DEPC-sensitive pH regulation of MCT4 function. However, His382 is not a direct substrate binding site because MCT4 activity did not disappear with this mutation.

**Table 2:** Kinetic parameters of lactate transport via MCT4.

	MCT4-WT	MCT4-H382A
***K*_m_ (mM)**	3.4±0.4*	**3.3±0.3**
***V*_max_ (pmol/min/oocyte)**	383±17*	**55±1.6**

*Taken from reference 1.

**Table 3:** pH regulation of wild-type and mutant MCT4 functions.

	Lactate uptake (pmol/min/oocyte)		
	pH 7.5	pH 5.5	-Fold (pH 7.5/pH 5.5)
**WT**	**3.0±0.4**	**12.9±0.5**	**0.2**
**WT/DEPC**	**1.5±0.3**	**1.5±0.4**	**1.0**
**H382A**	**2.8±0.2**	**2.8±0.2**	**1.0**

Data were taken from Figs 2B and 7D.

## Discussion

In this study, we found an MCT4-specific inhibitory effect of zinc at pH 7.5 on lactate transport *via* MCT4, indicating that the zinc-susceptible residue is one of the functional residues of MCT4. There was no inhibitory effect of zinc in an acidic condition (pH 5.5), suggesting that zinc could not bind to the residue in a protonation state. The p*K*_a_ value of this residue is between 5.5 and 7.5. This value accords with p*K*_a_ of the histidine residue. Moreover, it is known that the order of the strength of affinity to histidine is copper > nickel > zinc [17–19], in agreement with this experimental result. Under the experimental conditions used in our study, we could not detect any interaction of metal with MCT1. We assumed that the difference is because the histidine residues between MCT1 and MCT4 are not conserved.

Treatment with DEPC, a histidine-modifying reagent, inhibited lactate uptake *via* MCT4, but ~20% of MCT4 activity was retained. To obtain further evidence for the role of histidine residues, the influence of DEPC on pH dependency of MCT4 function was investigated. The pH regulation of transport activity completely disappeared with DEPC treatment in MCT4-expressing oocytes, suggesting that the DEPC-sensitive histidine residue is an extracellular pH sensor for regulation of MCT4 function. We tried DEPC modification, but MCT1 activity was abolished and investigation of the transport mechanism was therefore impossible.

In the next series of experiments, the effect of DEPC modification in the presence of monocarboxylic acids and zinc was tested. We found that DEPC modification in the presence of lactate as a classical substrate for MCT4 preserved the transport activity, indicating that the important residues of histidine are protected by a substrate excess. We also found that the residue is protected by nicotinic acid but not by propionic acid and monocarboxylate drugs such as salicylic acid and ibuprofen. These results indicate that propionic acid and the drugs may be not substrates for MCT4. In fact, we could not obtain evidence that MCT4 transports these compounds including propionic acid, salicylic acid and ibuprofen. Although both nicotinic acid and lactate were shown to be MCT4 substrates, the effect of nicotinic acid was only half of that of lactate. The reason for this may be that the water solubility and affinity of nicotinic acid are low [20, 21]. Emoto *et al*. reported that nonsteroidal anti-inflammatory drugs (NSAIDs), such as salicylic acid and ibuprofen, noncompetitively inhibited the uptake of lactate [22]. Therefore, our data lend support to the results of the previous study showing that a lactate transporter does not have an NSAID transport function. However, Hosoya *et al*. reported that rat lactate transporter-mediated lactate uptake was inhibited by salicylic acid, which competitively inhibited this process [23]. The functions of human MCT isoforms and rat Mct isoforms may be different. The DEPC-induced suppression of lactate uptake *via* MCT4 was also prevented by zinc. These results indicated that at least one DEPC-sensitive histidine residue located near the substrate binding site is responsible for conferring pH regulation. It is well known that metals are combined with a protein surface. For instance, it has been reported that zinc inhibits proton-dependent glycine transport by glycine transporter subtype 1b [24]. This transporter has histidine residues in the extracellular loops and these residues are zinc-binding sites.

**Fig 8:**
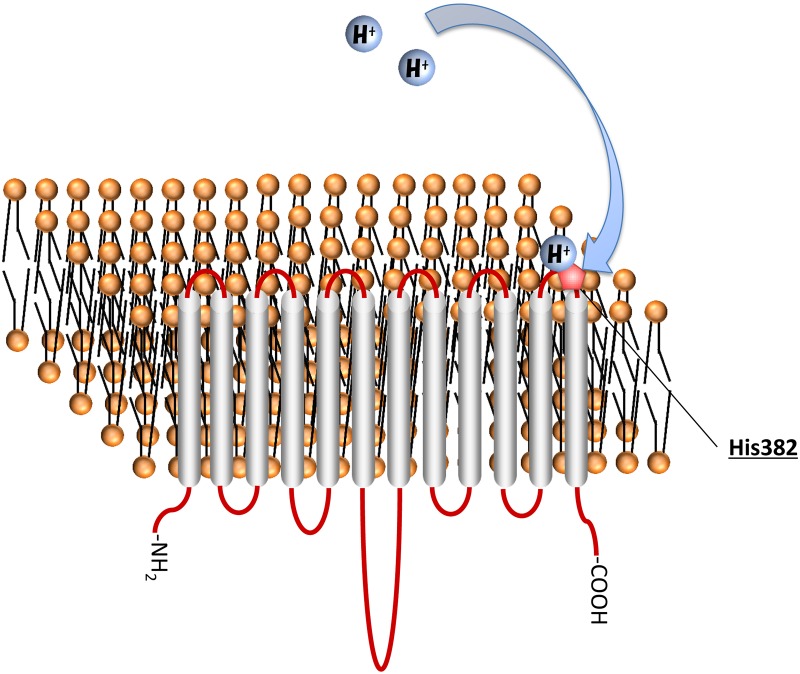
His382 is extracellular pH sensor. Taken together, our findings demonstrate that the non-conserved residue His382 in the extracellular loop of MCT4 is essential for pH regulation of MCT4-mediated lactate transport activity. The results of this study should be useful for development of a drug delivery system using MCT4.

In order to determine whether histidine residues are involved in the regulation of transport activity by pH, we mutated an amino acid residue that might be involved in MCT4-mediated lactate transport. MCT4 has five histidine residues, one in the extracellular loop, another in the intracellular loop and the other three in the carboxy terminal facing the cytosol. It is very unlikely that histidine residues on the intracellular side participate in the pH regulation of transport function. Hence, we analyzed the role of His382 for pH dependency in the function of MCT4. On switching from pH 5.5 to 7.5, pH 7.5/pH 5.5 uptake ratio of MCT4-WT was 0.2 (Table 3). On the other hand, the ratios of MCT4-WT/DEPC and MCT4-H382A were 1.0 and 1.0, respectively. It is known that DEPC cannot react with histidine residues located in the intracellular side within membrane proteins. Therefore, the possibility that histidine residues located in the intracellular side contribute to extracellular pH-dependent lactate uptake *via* MCT4 is very low. Intracellular pH dependency of MCT4-mediated efflux has not been reported. Histidine residues other than His382 (His350, His430, His445 and His459) might be relevant to intracellular pH-dependent lactate efflux *via* MCT4. Interaction of between His382 and lactate was not detected, suggesting that His382 is not a substrate binding site because the *K*_m_ value for MCT4-mutant was similar to *K*_m_ value for MCT4-WT. In addition, Zinc and DEPC did not affect transport in the mutant in which MCT4 activity had been significantly suppressed. Therefore, it is thought that the histidine residue is not a part of the substrate binding pocket and, as a consequence, the most probable histidine residue seems to be the proton coupling site, His382. We have already reported that Arg278 is involved in lactate recognition by MCT4 [1]. Hence, Arg278 and His382 in the structure of MCT4 probably exist at nearby locations. We also examined the potential interaction of a proton with this residue. It is known that histidine residues are involved in binding and movement of protons by other carriers [11, 25, 26] and may operate as a pH sensor [27]. The pH regulation of MCT4-H382A function was knocked out completely. It seems that this residue is involved in binding and movement of a proton (Fig 8).
